# Acceptability of safe drug consumption spaces among people who inject drugs in rural West Virginia

**DOI:** 10.1186/s12954-019-0320-8

**Published:** 2019-08-31

**Authors:** Allison O’Rourke, Rebecca Hamilton White, Ju Nyeong Park, Kayla Rodriguez, Michael E. Kilkenny, Susan G. Sherman, Sean T. Allen

**Affiliations:** 1 DC Center for AIDS Research, Department of Psychology, George Washington University, 2125 G St. NW, Washington, DC, 20052 USA; 2 Department of Health, Behavior and Society, Johns Hopkins University Bloomberg School of Public Health, 624 N. Broadway, Baltimore, MD 21205 USA; 3 Joan C. Edwards School of Medicine, Marshall University, 1249 15th Street, Huntington, WV 25701 USA; 4 Cabell-Huntington Health Department, 703 7th Ave, Huntington, WV 25701 USA

**Keywords:** People who inject drugs, Rural public health, Harm reduction, Supervised consumption spaces, Supervised injection facilities, PWID

## Abstract

**Aim:**

Safe consumption spaces (SCS) are indoor environments in which people can use drugs with trained personnel on site to provide overdose reversal and risk reduction services. SCS have been shown to reduce fatal overdoses, decrease public syringe disposal, and reduce public drug consumption. Existing SCS research in the USA has explored acceptability for the hypothetical use of SCS, but primarily among urban populations of people who inject drugs (PWID). Given the disproportionate impact of the opioid crisis in rural communities, this research examines hypothetical SCS acceptability among a rural sample of PWID in West Virginia.

**Methods:**

Data were drawn from a 2018 cross-sectional survey of PWID (*n* = 373) who reported injection drug use in the previous 6 months and residence in Cabell County, West Virginia. Participants were asked about their hypothetical use of a SCS with responses dichotomized into two groups, likely and unlikely SCS users. Chi-square and *t* tests were conducted to identify differences between likely and unlikely SCS users across demographic, substance use, and health measures.

**Results:**

Survey participants were 59.5% male, 83.4% non-Hispanic White, and 79.1% reported likely hypothetical SCS use. Hypothetical SCS users were significantly (*p* < .05) more likely to have recently (past 6 months) injected cocaine (38.3% vs. 25.7%), speedball (41.0% vs. 24.3%), and to report preferring drugs containing fentanyl (32.5% vs. 20.3%). Additionally, likely SCS users were significantly more likely to have recently experienced an overdose (46.8% vs. 32.4%), witnessed an overdose (78.3% vs. 60.8%), and received naloxone (51.2% vs. 37.8%). Likely SCS users were less likely to have borrowed a syringe from a friend (34.6% vs. 48.7%).

**Conclusions:**

Rural PWID engaging in high-risk behaviors perceive SCS as an acceptable harm reduction strategy. SCS may be a viable option to reduce overdose fatalities in rural communities.

## Introduction

In 2017, there were more than 70,000 overdose fatalities in the USA (21.7 deaths per 100,000 people) with the highest state overdose mortality rate in West Virginia (57.8 deaths per 100,000 people) [1]. No area of West Virginia has been harder hit by the opioid crisis than Cabell County which has the highest overdose mortality rate in the state reporting 152 deaths in 2017 and accounted for more than 20% of overdose fatalities in the state [2]. In addition to overdose, people who inject drugs (PWID) are also at increased risk for HIV, hepatitis C (HCV), and skin and soft tissue infections [3–6]. In the wake of the 2015 Scott County, Indiana HIV epidemic where 215 incident cases of HIV were linked to injection drug use [7], 220 counties were identified as vulnerable to similar HIV outbreaks [8]. These counties were concentrated in the Appalachian region of the eastern US; for example, 28 of the 55 counties in West Virginia were identified as vulnerable. Risks for adverse health consequences associated with the modern opioid crisis are exacerbated by multiple structural vulnerabilities (e.g., homelessness, poverty, diminished access to HIV prevention services) that are highly prevalent among PWID populations, especially those residing in rural areas. Additionally, PWID that are structurally vulnerable may also engage in public drug use, which has been linked to several adverse outcomes, including overdose, syringe sharing, and discarded syringes [9–14]. As rural communities respond to the consequences of the opioid crisis, many have chosen to implement harm reduction programs to not only prevent overdose fatalities and infectious disease outbreaks, but also link PWID to drug treatment programs and other essential health and human services.

Safe consumption spaces (SCS), also known as overdose prevention sites, supervised injection facilities, and drug consumption spaces, are public health facilities where individuals can use drugs under the supervision of trained staff [15]. SCS offer sterile injection supplies (e.g., syringes, tourniquets, cookers) and proper disposal containers for waste related to injection drug use. Additionally, many SCS also provide naloxone, drug counseling, referrals to medical care and addiction treatment, HIV and HCV testing, and other social services. There are over 90 of these sites operating across the globe. The first SCS to open in North America was Insite, in Vancouver, Canada, in 2003 [16]. Research has shown that SCS reduce overdose fatalities, public injection, HIV transmission, and publicly discarded syringes and injection-related litter (e.g., cookers) while increasing enrollment in addiction treatment services [17–24]. For example, in a study examining the overdose mortality rates outside of an SCS in Vancouver in the 2 years after its opening, researchers identified a 35% decrease in overdose mortalities within approximately one third of a mile of the SCS compared to 9% during the same period in the rest of the city [25]. Further, SCS have been shown to be a cost-effective public health strategy via reducing treatment costs associated with incident HIV/HCV infections, wound care, and overdose-related services [26, 27]. Research has also shown that SCS implementation does not increase crime and can lead to reductions in calls for emergency services [28, 29]. A research study in Sydney, Australia examined calls to emergency responders for overdose and found an 80% reduction in calls in the area immediately surrounding the SCS during operating hours and a 45% reduction in calls across the broader surrounding area [30].

A small number of research studies have examined hypothetical acceptability of SCS among PWID in the US [31–34]. These studies report between 63 and 92% of PWID indicated likely use of a SCS if one existed in their community. Intent to use a hypothetical SCS was found to be most acceptable among those individuals reporting high-risk drug use behaviors (e.g., having recently used fentanyl or experienced an overdose), those engaging in public drug use, and those reporting being homeless. Current published studies were all conducted in urban settings; less is known about hypothetical SCS acceptability among PWID in rural areas. Given the disproportionate impact of the opioid crisis in rural communities, this research examines the hypothetical acceptability of SCS utilization among a rural sample of PWID in West Virginia.

## Methods

This research is part of a larger study that aimed to estimate the number of PWID in Cabell County, WV; detailed methods describing data collection in the parent study can be found in related publications [35–37]. For brevity, we provide an overview. Surveys were conducted in June and July 2018 in Cabell County, West Virginia, during two separate, 2-week data collection periods as part of a capture-recapture population estimation study. Surveys were administered via audio computer-assisted self-interview (ACASI) at the Cabell-Huntington Health Department (CHHD) harm reduction program and in areas throughout the county where PWID frequent, such as local transportation hubs (i.e., bus stops), parks, and gas stations. The CHHD harm reduction program operates a single fixed site location which is open during normal health department operating hours and offers syringe exchange, naloxone, HIV and HCV testing, and referrals to drug treatment. Participants were verbally consented and received either a $10 grocery gift card or a small bag with food items as an incentive. In total, 373 individuals taking the survey indicated injection drug use in the past 6 months and residing in Cabell County, West Virginia. The institutional review board at the Johns Hopkins Bloomberg School of Public Health approved this research.

## Measures

To assess hypothetical acceptability of SCS use, survey participants were first given a description of a SCS, “We want to ask you a question about a safe consumption space for drug use. A safe consumption space is a place where it would be legal for people to safely inject, snort, or smoke, or otherwise consume drugs that they buy somewhere else. You would not be arrested while in the site. There would be staff on site to respond to an overdose, and to provide basic medical care and referrals to health and social services upon request.” They were then asked, “How likely is it that you would use a safe consumption space?” with response options on a 4-point Likert scale from very likely to very unlikely. For these analyses, responses were dichotomized into “likely” (somewhat likely or very likely) and “unlikely” (somewhat unlikely or very unlikely) SCS users.

Socio-demographics included in these analyses were age, gender (male or female), race, ethnicity, sexual orientation, relationship status, education, homelessness, arrest, and current health insurance. Age was reported as a continuous measure. Due to a lack of diversity in responses related to race and ethnicity, these measures were collapsed to White, non-Hispanic versus all others. Relationship status was dichotomized to those currently married or with a partner vs. divorced or single. Education was dichotomized to those having graduated from high school or equivalent (GED) and those with education less than a high school diploma. Homelessness was assessed by participant yes/no responses to the question “Do you consider yourself homeless?”. Sexual minority status was defined as all individuals who identified as gay, lesbian, bisexual, or other orientation. Arrest was self-reported for any arrest in the previous 6 months.

Drug use measures included years of injection drug use, recent (past 6 months) drugs injected, and preference for drugs containing fentanyl. Years of injection drug use was calculated using reported age at time of survey administration and age at first injection drug use. Injected drug categories included cocaine, heroin, speedball (heroin and cocaine), crystal methamphetamine, fentanyl, buprenorphine or suboxone, and painkillers.

Injection drug use behaviors were measured by where they had recently (past 6 months) received sterile syringes, receptive injection equipment sharing, public drug consumption, overdose experiences, and naloxone access. Participants endorsed possible locations in which they had received sterile syringes in the past 6 months, including borrowed from a friend, a needle exchange program, and bought/received from another person. The number of recent (past 6 months) overdose experiences (fatal and nonfatal) witnessed were reported as continuous measures. For these analyses, responses were collapsed to a single variable indicating if a participant had witnessed at least one overdose (fatal or nonfatal) in the past 6 months. For having experienced a recent overdose, continuous responses were similarly dichotomized. Having recently (past 6 months) received naloxone was reported dichotomously (yes/no) via “…did you get Narcan or naloxone from any sources to prevent an overdose?.” Public drug consumption was dichotomized as those reporting typical recent (past 6 months) drug use location as in a home (their own or that of a friend) versus all other locations. Other locations included on the street, at a park, a stairwell in a building, abandoned building, public transit location (bus, train, etc.), a public bathroom, in the woods, and in a vehicle. Receptive injection equipment sharing was measured as reporting any use of syringes, cookers, cottons, or rinse water that the participant knew had been used by someone else during the previous 6 months.

Health-related measures included self-reported recent (past 6 months) HIV and HCV testing, wanting drug treatment, and comfort discussing drug use with doctors. For recent interest in wanting drug treatment, participants were asked “In the past 6 months, have you wanted to start drug treatment but not been able to get into a program?” and responded dichotomously (yes/no). Participants were asked how comfortable they would be talking to their doctor about their drug use and responded on a 4-point Likert scale from very comfortable to very uncomfortable. Responses were dichotomized to comfortable (very comfortable and somewhat comfortable) and uncomfortable (very uncomfortable and somewhat uncomfortable) talking about their drug use.

Differences between the likely and unlikely SCS group were analyzed across socio-demographics, drug use, injection drug use behaviors, and health-related measures using chi-square and *t* tests in SAS 9.4.

## Results

Nearly 80% (*n* = 295) of rural PWID reported very likely or likely use of a SCS. Table 1 summarizes the statistical findings.

**Table 1:** Characteristics of a rural population of PWID by hypothetical usage of a safe consumption space (SCS)

Characteristic	Overall (*N* = 373), *N* (%)	Likely SCS users (*n* = 295), *N* (%)	Unlikely SCS users (*n* = 74), *N* (%)	Chi-square/*t* test, *P* value
Age (mean, SD)	35.8 (8.6)	35.8 (8.3)	35.6 (10.0)	.8536
Male	222 (59.5)	173 (58.6)	47 (63.5)	.4453
White, non-Hispanic	302 (83.4)	243 (84.7)	58 (79.5)	.2823
Sexual minority	64 (17.1)	53 (18.0)	11 (14.9)	.5287
Currently in a relationship	175 (47.3)	140 (47.5)	35 (47.3)	.9803
High school graduate or equivalent	266 (71.7)	209 (70.9)	56 (75.7)	.4091
Consider self-homeless	213 (57.1)	171 (58.0)	41 (55.4)	.6904
Health insurance	273 (73.2)	217 (73.6)	52 (70.3)	.5692
Arrest*	114 (30.6)	95 (32.2)	18 (24.3)	.1886
Years of injection drug use (mean, SD)	10.9 (9.2)	11.3 (9.2)	9.5 (9.3)	.1491
Drugs injected*				
Heroin	306 (82.0)	244 (82.7)	60 (81.1)	.7420
Crystal methamphetamine	265 (71.0)	214 (72.5)	49 (66.2)	.2822
Fentanyl	210 (56.3)	168 (57.0)	40 (54.1)	.6534
Speedball	141 (37.8)	121 (41.0)	18 (24.3)	.0081
Cocaine	132 (35.4)	113 (38.3)	19 (25.7)	.0427
Buprenorphine or Suboxone	111 (29.8)	90 (30.5)	20 (27.0)	.5583
Pain killers	81 (21.7)	65 (22.0)	14 (18.9)	.5592
Prefer fentanyl in drugs*	112 (30.1)	96 (32.5)	15 (20.3)	.0396
Places received sterile syringes*				
Borrowed from a friend	139 (37.3)	102 (34.6)	36 (48.7)	.0253
Needle exchange program	246 (66.0)	200 (67.8)	46 (62.2)	.3579
Bought/received from another person	132 (35.4)	98 (33.2)	32 (43.2)	.1066
Any receptive injection equipment sharing*	225 (60.3)	177 (60.0)	47 (63.5)	.5800
Public drug use*	175 (48.6)	144 (50.7)	29 (40.3)	.1139
Experienced a drug overdose*	163 (43.7)	138 (46.8)	24 (32.4)	.0262
Witnessed a drug overdose*	267 (74.3)	231 (78.3)	45 (60.8)	.0019
Received naloxone*	179 (48.3)	151 (51.2)	28 (37.8)	.0399
Comfortable talking to doctor about drug use	251 (68.0)	208 (71.0)	41 (55.4)	.0103
Wanted drug treatment but unable to get into a program*	134 (36.3)	113 (38.4)	21 (28.8)	.1246
HIV test*	194 (52.3)	162 (54.9)	32 (43.2)	.0722
Hepatitis C test*	193 (52.0)	161 (54.6)	32 (43.2)	.0809

*Reported based on last 6 months

Demographically, PWID were 35.8 years old (SD 8.6) and primarily male (59.5%), White, non-Hispanic (83.4%), and had at least a high school education (71.7%). Almost half were either married or in a relationship (47.3%). Socio-demographic measures of PWID showed 73.2% reported currently having health insurance, 57.1% reporting considering themselves homeless, and 30.6% reported having been recently arrested. No significant differences were found in socio-demographic measures between likely and unlikely SCS users.

The mean number of years of injection drug use averaged 10.9 (SD 9.2) years. The most commonly reported recently injected drugs were heroin (82.0%), crystal methamphetamine (71.0%), and fentanyl (56.3%). Likely SCS users reported significantly more recent injection use of speedball (cocaine and heroin) (41.0% vs. 24.3%, *p* = .0081) and cocaine (38.3% vs. 25.7%, *p* = .0427). Likely SCS users were significantly more likely to prefer drugs containing fentanyl than unlikely SCS users (32.5% vs. 20.3%, *p* = .0396).

When reporting places where PWID recently received new, sterile syringes, needle exchange programs were most commonly reported (66.0%) followed by borrowing from a friend (37.3%). Likely SCS users were significantly less likely to report having borrowed a syringe from a friend than unlikely SCS users (34.6% vs. 48.7%, *p* = .0253). Receptive injection equipment sharing (syringes, cookers, rinse water, or cotton) was reported by 60.3% and public drug use was reported by 48.6% of PWID.

Significant differences were found between likely and unlikely SCS users on measures of recent drug-related experiences. Significantly more likely SCS users reported recently experiencing an overdose (46.8% vs. 32.4%, *p* = .0262), witnessing a recent overdose (78.3% vs. 60.8%, *p* = .0019), and having recently received naloxone (51.2% vs. 37.8%, *p* = .0399).

In the area of health behaviors, likely SCS users reported being significantly more comfortable talking to doctors about their drug use than unlikely SCS users (71.0% vs. 55.4%, *p* = .0103). Additionally, though not significantly different from their unlikely SCS user counterparts, likely SCS users were more likely to report having recently (past 6 months) wanted drug treatment but not being able to get into a program (38.4% vs. 28.8%, *p* = .1246), received an HIV test (54.9% vs. 43.2%, *p* = .0722), and received a hepatitis C test (54.6% vs. 43.2%, *p* = .0809).

## Discussion

To our knowledge, this is the first study that has examined hypothetical SCS acceptability among a rural population of PWID, thus building on previous research in urban settings. In our study, PWID who reported likely SCS utilization reported being at significantly greater risk of overdose; for example, SCS users reported higher rates of recently witnessing an overdose, experiencing an overdose, injecting cocaine and speedball (cocaine and heroin), and preferring drugs containing fentanyl. Hypothetical SCS users were also more likely to already be engaging in harm reducing behaviors (e.g., HIV and HCV testing, less syringe borrowing) compared with their unlikely SCS utilizing counterparts. Lastly, likely SCS users reported significantly higher levels of comfort talking about their drug use with their doctors and wanting to get into drug treatment (but unable to get into a program). Our findings highlight that the portion of the PWID population that is more likely to use a hypothetical SCS reflects persons who would likely benefit the most from SCS utilization (i.e., those engaging in higher-risk injection drug use behaviors).

Our finding that nearly 80% of rural PWID reported likely hypothetical SCS utilization was similar to acceptability levels found in urban PWID populations in the USA (63 to 92%) [31–34]. Because data in our analyses were collected as part of a PWID population estimation involving the recruitment of individuals from a variety of locations in the community, findings are more likely to reflect the broader community-level acceptability of hypothetical SCS utilization among the PWID population in Cabell County. The high level of hypothetical SCS acceptability is also noteworthy given the stigmatization of addiction in rural communities. Unlike metropolitan areas, rural communities have experienced significant struggles to implement evidence-based opioid crisis response strategies (e.g., medication-assisted treatment, syringe services programs). Recent qualitative research indicates that rural PWID are regularly subjected to stigmatizing language, behaviors, and policies that impede their ability to engage in positive health-seeking behaviors [38]. Given that SCS implementation in rural areas would require community support, future research should be conducted to understand overall community-level awareness of and acceptability for SCS implementation as well as where facilities could be implemented that best serve geographically dispersed PWID populations.

In comparison to related SCS acceptability research in the USA, we found both similarities and differences. Compared with urban-based SCS acceptability research, we did not find homelessness or public drug consumption to be related to likely SCS use, despite high levels of these indicators among our sample. Paralleling urban-based SCS acceptability research, we did, however, find that likely SCS users were more likely to report fentanyl preference and high rates of recent overdose experiences. Additionally, studies in urban settings have found that attendance at SCS has led to increased enrollment in drug treatment. Among our population who reported wanting drug treatment (but unable to get into a program), significantly more reported likely hypothetical SCS use, indicating that SCS implementation may be another avenue for connecting rural PWID to drug treatment programs. Given that our data are only able to speak to hypothetical SCS acceptability among a single rural PWID population, comparable research is needed in other non-urban areas to fully understand how urban and rural areas may differ in SCS acceptability.

Combining our hypothetical SCS acceptability data with related research allows us to estimate potential SCS utilization if a facility opened. In a study by DeBeck et al. [39], 72% of PWID who indicated willingness to use a SCS later accessed the SCS when it opened. Based on a recent study that estimated there were 1857 (95% CI 1147, 2657) PWID in Cabell County [33], we estimate that 1469 (95% CI 907, 2102) PWID would be open to using a SCS and 72% of that population (approximately 1058 [95% CI 653, 1513]) would potentially utilize the facility, if it existed. These data demonstrate that there is a large number of people in West Virginia that may potentially benefit from SCS implementation and that there is a need for additional research to understand how these programs could best serve rural PWID at high risk of HIV and overdose.

Cabell County has the highest drug overdose, fatal and nonfatal, rates in WV, and stakeholders throughout the community (e.g., first responders, Cabell-Huntington Health Department, local faith-based leaders, substance use treatment organizations) have joined forces to implement collaborative initiatives designed to improve the public health of PWID. These efforts have shown preliminary success; for example, compared to 2017, there was a 40% reduction in EMS calls for overdose in 2018 [40]. Even with these measures, the overdose rate remains very high and continues to tax the community’s resources. One particularly high-risk period for overdose among PWID is in the time immediately following when persons are released from incarceration. Our findings indicate that close to one third of our participants reported having been recently arrested. Having an SCS may serve as a venue for PWID reentering the community to engage in harm reduction service provision and be connected to drug treatment programs as they reintegrate into society. SCS implementation may positively affect rural communities via reducing overdose fatalities, HIV incidence, and discarded syringes; however, additional research is warranted as SCS are both hypothetical interventions in the US and their implementation may differ between urban and rural contexts.

Our study had several limitations. First, because SCS implementation in a US context remains hypothetical, asking PWID about their utilization may conjure very different images across our study population. Further research should be undertaken to understand how rural communities, particularly first responders and public health officials, would envision a SCS operating in their community and how this vision marries that of PWID. Second, injection drug use is highly stigmatized, especially in rural communities. As such, it is possible that some PWID did not take the survey in order to avoid stigmatization. Last, social desirability bias may have impacted how participants answered survey questions. We attempted to mitigate this bias by administering the survey via ACASI and collecting data anonymously. Despite these limitations, the study did reach a very diverse group of rural PWID and provides valuable insights into the characteristics of the population who likely would use a SCS.

## Conclusion

Acceptability of hypothetical SCS use was very high among our rural population of PWID in WV. Our findings suggest that rural PWID most likely to use a hypothetical SCS also engage in high-risk injection drug use practices. Given that SCS implementation remains a hypothetical scenario in a US context, future research should qualitatively explore factors associated with SCS acceptability among rural PWID. SCS may be a viable option to reduce overdose fatalities in rural communities.

## Data Availability

The datasets generated and/or analyzed during the current study are not publicly available due to concerns about confidentiality.

## Abbreviations

HCV: hepatitis C
HIV: human immunodeficiency virus
IDU: injection drug use
PWID: people who inject drugs
SCS: safe consumption spaces
USA: United States of America
WV: West Virginia
